# Fundamental limits and design principles of doublet metalenses

**DOI:** 10.1515/nanoph-2021-0770

**Published:** 2022-02-16

**Authors:** Augusto Martins, Juntao Li, Ben-Hur V. Borges, Thomas F. Krauss, Emiliano R. Martins

**Affiliations:** São Carlos School of Engineering, Department of Electrical and Computer Engineering, University of São Paulo, São Carlos 13566-590, Brazil; State Key Laboratory of Optoelectronic Materials and Technologies, School of Physics, Sun Yat-sen University, Guangzhou 510275, China; School of Physics, Engineering and Technology, University of York, York YO10 5DD, UK

**Keywords:** diffraction limited focusing, doublets, metalenses, wide field of view

## Abstract

Metalenses are nanostructured surfaces with great potential for delivering miniaturized and integrated optical systems. A key property of metalenses is that, by using a double layer configuration, or doublet, they can achieve both diffraction-limited resolution and wide field-of-view imaging. The physical operation and limitations of such doublet systems, however, are still not fully understood, and designs are still based on numerical optimization of the phase profiles. Here, we show the fundamental limits of doublet systems and provide a universal design strategy without any need to resort to numerical optimization. We find an analytical relationship between the focal length and the spacer thickness; we identify the physical principles underlying the limitations on performance and obtain a universal dependence of the field of view as a function of resolution (numerical aperture). Our results will allow researchers to appreciate the regimes of resolution and field of view that are accessible for specific applications, to identify the conditions for optimum performance (such as required spacer thickness), and to conveniently design doublets without needing to resort to numerical optimizations.

## 1 Introduction

Metalenses are an emerging technology that holds great promise to deliver miniaturized and lightweight optical systems at a low cost [1], [2], [3], [4]. A major advantage of metalenses is their versatility in modulating an optical beam by appropriately engineering their constituent meta-atoms, especially for the generation of arbitrary phase profiles. Such versatility is explored in a number of applications, such as diffraction-limited focusing [5], [6], [7], [8], [9], achromatic focusing [10], [11], [12], [13], [14], [15], [16], [17], [18], [19], Stokes cameras [20], and endoscopic optical systems [21, 22], to mention but a few. A major challenge of many high-resolution metalenses, however, is their limited field-of-view (FOV) due to off-axis aberrations [23], [24], [25]. It is clear from fundamental principles that the only way to improve the FOV of diffraction-limited metalenses is through a double-layer configuration, or doublet [4]. Such a requirement is an instance of the well-known principle of optical systems that the simultaneous correction of off-axis and spherical aberrations requires more than one surface. Doublet metalenses were introduced by Arbabi et al. [24], and since then have been applied to important optical systems [16, 24], [25], [26], [27], such as cameras [24, 25] and endoscopic imaging systems [22]. Doublets have even been used to obtain different functionalities such as adjustable focal length systems [28, 29] and chromatic aberration correction [16, 18, 29]. Thus, doublets are not only a promising strategy for obtaining diffraction-limited resolution and wide FOV but are, in fact, a necessary requirement. The physical principles and fundamental limitations of doublet systems, however, are still not well understood. Indeed, current doublet designs are still widely based on numerical optimizations of the phase profiles, which must be performed anew for each specific system. Importantly, designs based on numerically obtained phase profiles can reach the optimum condition only for its designed configuration (for example, for a given spacer thickness and refractive index). Thus, without a more fundamental understanding of the physics of doublets, it is difficult to identify design strategies beyond phase engineering to further improve the systems’ performance. Therefore, it is necessary to understand the relevant physical principles and, particularly, to identify *a priori* the ranges of resolutions and fields-of-view achievable for specific applications.

Here, we describe the fundamental limitations of doublet systems, show that the spacer defines the focal length of the doublet, and elucidate the physical origin of the trade-off between resolution and FOV. Our analysis will allow researchers to understand the range of resolution and fields-of-view that can be obtained in doublet systems and easily design them for applications based on specific requirements.

## 2 Physical principles of doublet operation

**Figure 1:**
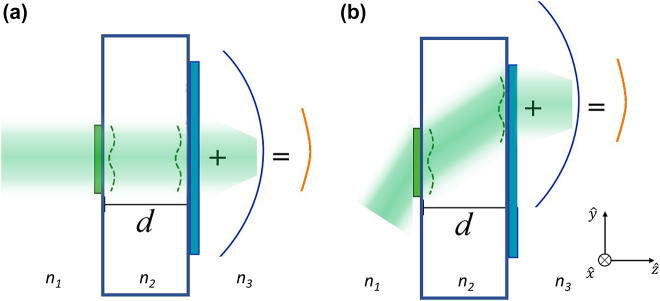
Pictorial representation of the doublet system operating at normal (a) and oblique (b) incidence. In both cases, the green dashed lines represent the phase profile of the Schmidt plate, while the blue and yellow solid lines represent the quadratic and hyperbolic phase profiles, respectively. In (b), the linear phase profile from the incidence was added to the quadratic phase profile at the output (drawn vertically displaced for that reason).

We begin with a qualitative description of the operation of a doublet. As shown in Figure 1, a doublet consists of two metasurfaces separated by a distance *d*. The first metasurface acts as a Schmidt plate (SP) by imposing a correcting phase, while the second metasurface imparts a quadratic phase profile. We define $\phi_{SP}$ as the phase modulation of the beam propagating from the first metasurface (the SP), and evaluated just behind the second (quadratic) metasurface. The phase imposed by the latter is defined as $\phi_{q}$ so the total phase imposed by the doublet is $\phi_{SP}+\phi_{q}$ . The operation of the doublet relies on a unique property of quadratic phase profiles, namely, the shifting of the coordinate axes at oblique incidence [30], [31], [32], [33], [34]. This effect is represented in Figure 1, which contrasts a quadratic phase at perpendicular incidence (Figure 1(a), blue curve) with the phase at oblique incidence (Figure 1(b), blue curve). At perpendicular incidence, the SP phase is added to the quadratic phase, resulting in a hyperbolic phase, thus leading to diffraction-limited focusing. The same hyperbolic phase profile must be used at oblique incidence to improve the FOV. To meet this requirement, we need to combine the optical propagation through the spacer, which is usually made of low index materials, but in principle can be any material – including air (see Section S8 of the supporting information (SI)), with the coordinate shifting of the quadratic phase profile. As illustrated in Figure 1(b), the SP phase is projected onto the shifted quadratic phase, resulting in a hyperbolic phase profile and, consequently, diffraction limited focusing. The space *d* between the surfaces is an integral part of the system. Its role can be understood from a more systematic analysis, to which we now turn our attention.

The quadratic phase profile is given by [30], [31], [32], [33],

(1) $$\phi_{q}\left(x,y\right)=-\frac{\pi n_{3}}{\lambda_{0}f_{q}}r^{2}$$

where $\lambda_{0}$ and $f_{q}$ are the free space wavelength and quadratic focal length, respectively, $n_{3}$ is the refractive index of the focusing medium and $r\equiv \sqrt{x^{2}+y^{2}}$ the radial coordinate in the metalens plane. To obtain diffraction-limited resolution, the input Schmidt plate has to convert a quadratic phase profile into a hyperbolic phase profile [23, 35], which is given by:

(2) $$\phi_{hyp}\left(x,y\right)=-\frac{2\pi }{\lambda_{0}}n_{3}\left(\sqrt{r^{2}+f_{h}^{2}}-f_{h}\right)$$

where $f_{h}$ is the focal length of the hyperbolic profile. At normal incidence, the doublet system output phase profile is approximately given by:

(3) $$\phi_{out}\left(r\right)≅\phi_{SP}\left(r\right)+\phi_{q}\left(r\right)$$

where $\phi_{SP}\left(r\right)$ is the SP phase profile. This relation holds only approximately because of the diffraction experienced by the beam as it propagates through the spacer. (Although we note that this effect can be easily accounted for using Fourier optics principles, see Section S2 of the SI for more details).

It is apparent from Eqs. (1)–(3) that the SP phase should be given by the difference between the hyperbolic and quadratic profiles

(4) $$\phi_{SP}\left(r\right)\approx \phi_{hyp}\left(r\right)-\phi_{q}\left(r\right)$$

The exact expression for the Schmidt plate can be found in Section S2 of the SI.

Next, we analyze the output phase profile at oblique incidence and highlight some constraints and limitations of the design. Oblique incidence is represented in Figure 1(b). If the Schmidt plate has a small spatial frequency bandwidth, we can assume that it preserves its phase profile after propagating inside the substrate and that it is only vertically displaced with the beam (see Section S3 of the SI for a rigorous demonstration). Note that the order of the metalenses depends on the desired application. If it is used as a telescope system as we demonstrate here, then the SP should be placed at the entrance. However, due to time reversal symmetry, it can also be used as an objective lens with the quadratic metalens placed at the entrance. Additionally, the oblique incidence carries a linear phase profile ( $yk_{0}n_{1}\;\sin\;\theta$ , where $k_{0}=\frac{2\pi }{\lambda_{0}}$ is the free space wavenumber and *θ* is the angle of incidence). When we combine this linear phase profile with the quadratic phase at the output, the net effect is a vertical displacement of the quadratic phase by $\frac{f_{q}}{n_{3}}\sin\;\theta$ . This can be easily demonstrated by completing squares:

(5) $$\phi_{q}\left(x,y\right)+yk_{0}n_{1}\;\sin\;\theta =-\frac{\pi n_{3}}{\lambda_{0}f_{q}}\left(x^{2}+y^{2}\right)-yk_{0}n_{1}\;\sin\;\theta =\phi_{q}\left(x,y-\frac{f_{q}}{n_{3}}n_{1}\;\sin\;\theta \right)+\frac{k_{0}^{2}n_{1}^{2}}{2n_{3}}f_{q}{\sin\;}^{2}\;\theta$$

where $\phi_{0}=\frac{k_{0}^{2}n_{1}^{2}}{2n_{3}}f_{q}{\sin\;}^{2}\;\theta$ is a constant phase term. Therefore, the output phase profile, in this case, is given by

(6) $$\phi_{out}^{\prime }\left(x,y\right)≅\phi_{S}\left(x,y-d\;\tan\;\theta^{\prime }\right)+\phi_{q}\left(x,y-\frac{f_{q}}{n_{3}}n_{1}\;\sin\;\theta \right)+\phi_{0}$$

where θ is the angle of incidence and $\theta^{\prime }=\text{asin}\left(\frac{n_{1}}{n_{2}}\sin\left(\theta \right)\right)$ is the angle of refraction in the substrate. Thus, substituting Eq. (4) into (6), the output phase profile is given by

(7) $$\phi_{out}^{\prime }\left(x,y\right)≅\phi_{hyp}\left(x,y-d\;\tan\;\theta^{\prime }\right)-\phi_{q}\left(x,y-d\;\tan\;\theta^{\prime }\right)+\phi_{q}\left(x,y-\frac{f_{q}}{n_{3}}n_{1}\;\sin\;\theta \right)$$

According to Eq. (7), the output phase profile consists of a displaced hyperbolic profile and two quadratic phase profiles, and both are also displaced vertically. To obtain diffraction-limited focusing, we need $\phi_{out}^{\prime }\left(x,y\right)≅\phi_{hyp}\left(x,y-d\;\tan\;\theta^{\prime }\right)$ , so the quadratic terms must cancel each other. Note that the first and second terms of Eq. (7), which come from the SP, are displaced by $d\;\tan\;\theta^{\prime }$ , and the third term by $\frac{f_{q}}{n_{3}}n_{1}\;\sin\;\theta$ . Thus, they can only vanish if the second surface is curved, that is, when:

(8) $$d=f_{q}\frac{n_{2}}{n_{3}}\cos\;\theta^{\prime }$$

where we used Snell’s law $n_{1}\;\sin\;\theta =n_{2}\;\sin\;\theta^{\prime }$ .

Thus, we have obtained a critical limitation on the FOV of a doublet system by removing the cosine function of Eq. (9) (see the Section S4 of the SI for more details), that is, by making the second metasurface planar instead of curved:

(9) $$d≅\frac{n_{2}}{n_{3}}f_{q}$$

Within this approximation, the output phase profile at oblique incidence reduces to a displaced hyperbolic profile:

(10) $$\phi_{out}^{\prime }\left(x,y\right)≅\phi_{hyp}\left(x,y-d\;\tan\;\theta^{\prime }\right)$$

Equations (1), (2), (4), and (9) are readily available to the designer (possibly with diffraction corrections, as described in Section S2 of the SI) without any need for numerical optimization. Note that the focal length of the doublet coincides with the focal length of the hyperbolic profile $f_{h}$ – as such, it is the distance between the focal point and the second (quadratic) metasurface, leaving the focal length of the quadratic phase $f_{q}$ as an additional degree of freedom. In the next section, we show how to choose $f_{q}$ to optimize the performance of the doublet.

### 2.1 The requirement of shallow phase modulation on the SP

As discussed in the previous section, the operation of the doublet system depends on the projection of the SP phase onto the quadratic phase. Since diffraction increases with modulation depth, it is necessary to minimize the phase modulation imposed by the SP to obtain the projection without distortion. This insight leads to an important design rule which establishes that to minimize the SP modulation; we need to control the focal length of the quadratic phase profile.

We begin the analysis that leads to this important design rule by noticing that, of all doublet parameters, only the quadratic lens focal length $f_{q}$ , the spacer thickness d , and the spacer refractive index $n_{2}$ do not directly affect the focal length of the doublet and its basic optical properties. With these three parameters related through Eq. (8), only two degrees-of-freedom are available to the design. The refractive index *n*_2_, however, is usually fixed by technological constraints, which leaves only one degree of freedom. Thus, we focus attention on the role of $f_{q}$ since this is the most easily controlled parameter. Our goal is to find the conditions that minimize the SP maximum spatial frequency modulus $(\max\left|\frac{\partial \phi_{SP}}{\partial r}|\right)$ , to reduce spurious diffraction in the spacer and preserve its phase at the output. As shown in Section S5 of the SI, given a doublet system with $F\equiv R_{a}/f_{h}$ , where $R_{a}$ is the SP radius, the optimum focal length ratio $\alpha =\frac{f_{q}}{f_{h}}$ that minimizes $\max\left|\frac{\partial \phi_{SP}}{\partial r}\right|$ is given implicitly by

(11) $$F\left(\alpha \right)={\left(\alpha^{\frac{2}{3}}-1\right)}^{\frac{1}{2}}\left[\alpha^{\frac{2}{3}}+{\left(\alpha^{\frac{4}{3}}+1-\alpha^{\frac{2}{3}}\right)}^{\frac{1}{2}}\right],\;\alpha \geq 1$$

**Figure 2:**
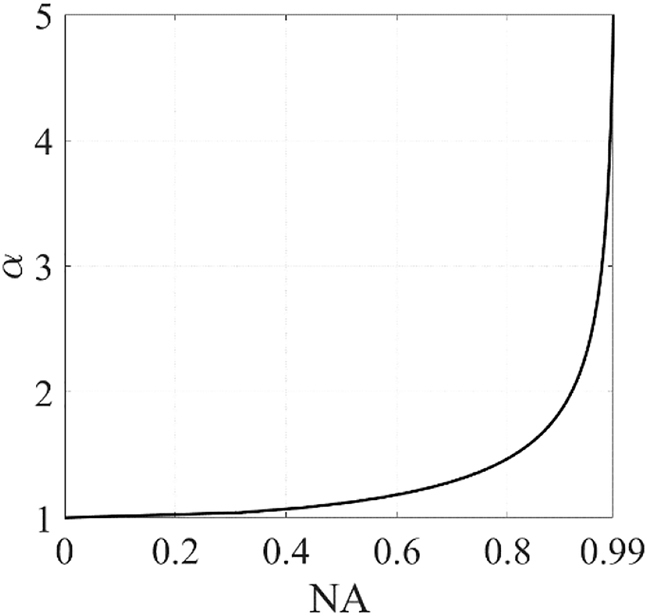
Optimum ratio between quadratic phase profile and hyperbolic phase profile $\left(\alpha =\frac{f_{q}}{f_{h}}\right)$ as function of the numerical aperture ( $NA\equiv \frac{F\left(\alpha \right)}{\sqrt{F{\left(\alpha \right)}^{2}+1}},$ with $F\left(\alpha \right)\equiv R_{a}/f_{h}$ and $R_{a}$ as the SP radius) that minimizes the phase gradient of the Schmidt plate.

In a diffraction limited system, such as the doublet, it is convenient to write the resolution in terms of the numerical aperture (NA), defined as $NA\left(\alpha \right)\equiv \sin\left(\text{atan}\left(F(\alpha \right)\right)=\frac{F\left(\alpha \right)}{\sqrt{F{\left(\alpha \right)}^{2}+1}}$ . The dependence of the focal length ratio α on the NA is shown in Figure 2. As α increases monotonically with the NA, for a fixed doublet focal length $f_{h}$ , a larger NA requires a larger $f_{q}$ . Interestingly, for a fixed *F* – or NA, the best ratio α depends neither on the doublet focal length nor on the other parameters. Importantly, the existence of an optimum ratio α stipulates that the doublet focal length $f_{h}$ depends on the spacer thickness through Eq. (8). In the next section, we analyze the performance of two doublets with the same NA but with different $f_{q}$ to understand the role of $f_{q}$ and how it affects the phase modulation imposed by the SP.

### 2.2 Design example

**Figure 3:**
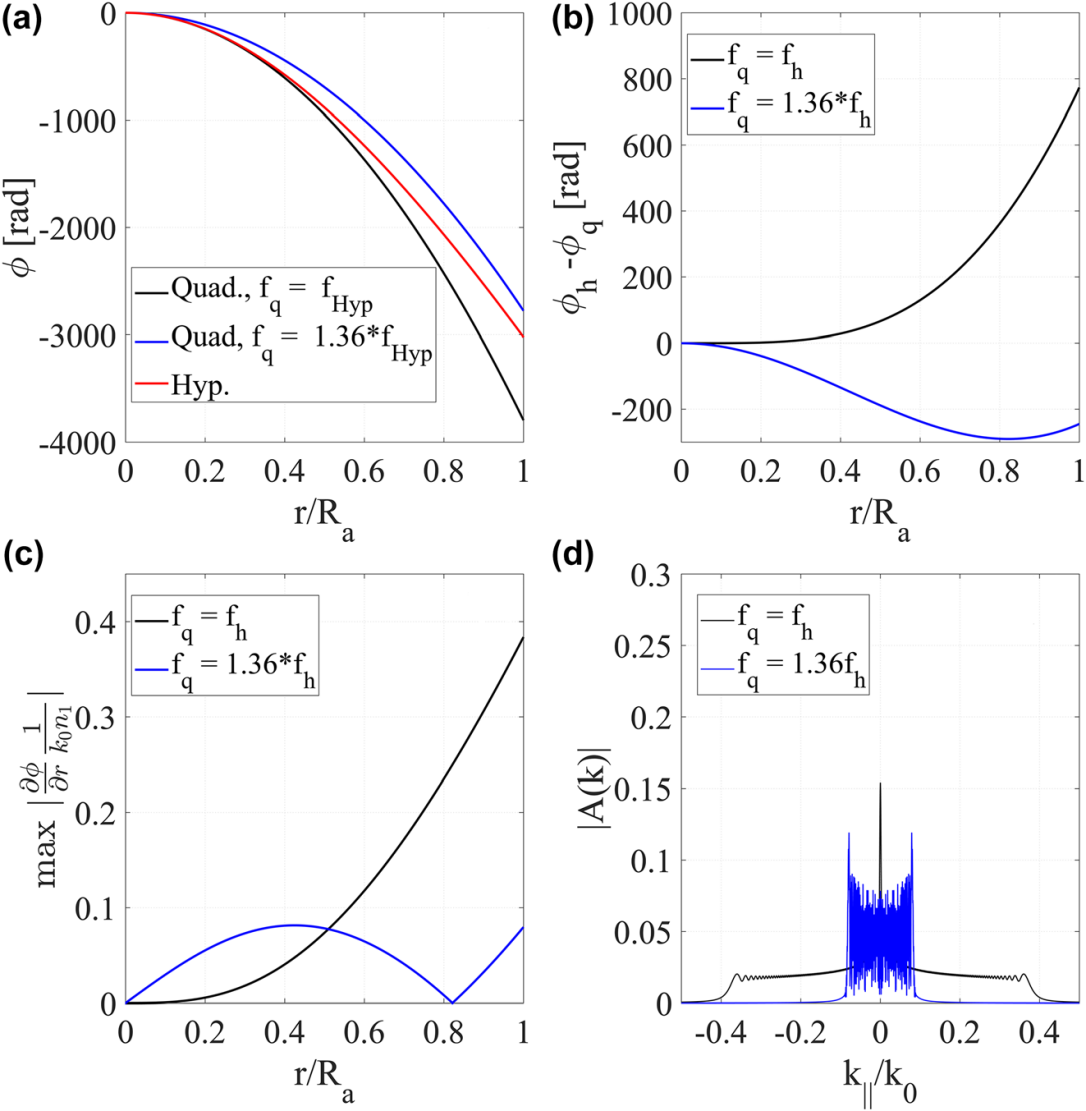
Doublet optimum design analysis. (a) Phase profile as function of the radial position. The red line refers to the hyperbolic phase profile with a focal length *f*_h_ = 100 μm , while the black and blue lines refer to the quadratic profile with focal lengths *f*_q_ = *f*_h_ and *f*_q_ = 1.36*f*_h_, respectively. (b)–(e) show, respectively, the approximated Schmidt plate phase profile, phase profile gradient modulus, and output field Fourier transform amplitude when *f*_q_ = *f*_h_ (solid black) and *f*_q_ = 1.36*f*_h_ (solid blue). The operating wavelength is 532 nm in all cases.

**Figure 4:**
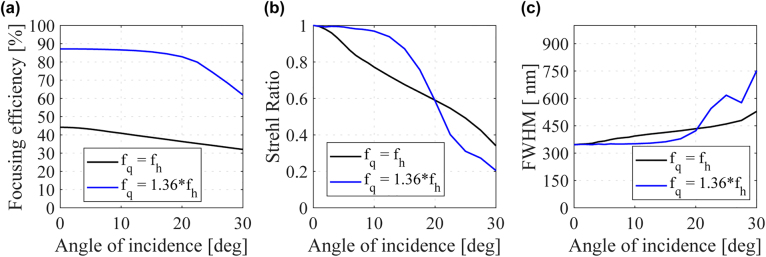
Doublet point spread function parameters as function of the angle of incidence for a quadratic metalens with a focal length equal to that of a hyperbolic metalens (solid black lines) and 1.36 times larger (blue solid lines). (a) Focusing efficiency, (b) Strehl ratio, and (c) FWHM. The operating wavelength is 532 nm, $f_{h}=100$ μm and NA = 0.75.

To illustrate how $f_{q}$ affects the spatial spectrum of the SP and, consequently, the doublet performance, we analyze two different scenarios: in the first, we set $f_{q}=f_{h}$ and in the second $f_{q}=\alpha f_{h}$ , with α given by the minimization condition of Eq. (11). For both cases, the remaining parameters are: *f*_h_ = 100 μm , *n*_1_ = *n*_3_ = 1, *n*_2_ = 1.45, *λ*_0_ = 532 nm. The spacer thickness is fixed by Eq. (8): $d=\frac{n_{2}}{n_{3}}f_{q}=1.45f_{q}$ . We also kept the numerical aperture fixed at NA = 0.75, which entails *R*_a_ = 113 μm . For this NA, the optimum α is 1.367 . Figure 3(a) shows a radial cut of the hyperbolic and quadratic phase profiles for both cases. As shown in Figure 3(a), when $f_{p}=f_{h}$ , the hyperbolic phase profile (red solid line) and the quadratic phase profile (black solid line) are close to each other in the short radius region but depart for larger radii [32]. Consequently, the SP phase profile – which is given by their difference according to Eq. (4) – increases very fast, as indicated by the black solid line in Figure 3(b). Since we are dealing with phase-only profiles, the local spatial frequency of the Schmidt plate is calculated as the radial derivative of the phase profile $\left(\frac{d\phi \left(r\right)}{dr}\right)$ [36]. The solid black line in Figure 3(c) shows the normalized absolute value of the local spatial frequency imposed by the SP when $f_{p}=f_{h}$ . Note that the maximum spatial frequency is 0.37 $\frac{\lambda_{0}}{2\pi }$ . Therefore, the transmitted field spatial spectrum (solid black line in Figure 3(d)) cuts off at approximately $k_{r}=0.37\frac{\lambda_{0}}{2\pi }$ . Such a broad spectrum leads to high diffraction in the spacer, thus deteriorating the performance of the doublet, as shown in Figure 4. To reduce the SP spectrum bandwidth, we need to increase the quadratic phase profile to its optimum condition $f_{p}=1.36f_{h}$ . Compare the quadratic and hyperbolic phase profiles for this case shown, respectively, by the blue and red curves in Figure 3(a). Notice that, since they have different focal lengths, the separation between the blue and red curves is larger than the separation between the black and red curves in the region of small radius ( $r/R_{a}<0.6$ ). For larger radii ( $r/R_{a}>0.6$ ), however, the tendencies are flipped, and now the separation between the blue and red curves is much smaller than the separation between the black and red ones. This phenomenon can be better observed when plotting their difference ( $\phi_{h}-\phi_{p},$ that is, the SP phase profile), shown as a comparison between the black and blue curves in Figure 3(b). Notice that, the phase difference increases monotonically for the non-optimum condition (black curve in Figure 3(b)), whereas the phase difference for the optimum condition (blue curve) reaches a minimum value at $r/R_{a}=0.8$ and then slightly increases again. This pattern was also observed in [25], where it received a ray optics interpretation. From a wave optics point of view, this oscillating pattern minimizes the SP phase gradient within the aperture region, as shown by the blue curve in Figure 3(c). In this case, the phase gradient modulus reaches a maximum of $k_{r}=0.08\frac{\lambda_{0}}{2\pi }$ for the optimum condition (blue curve in Figure 3(c)), in contrast with the monotonical increase for the non-optimum condition (black curve in Figure 3(c)). Such a difference translates into a bandwidth for the optimum condition (blue curve in Figure 3(d)) smaller than the non-optimum condition (black curve in Figure 3(d)).

To quantify the performance gain obtained with the optimum $f_{q}$ , we calculate the point spread functions (PSFs) for different angles of incidence using the angular spectrum formalism [36]. More details about the calculations are available in Section S1 of the SI. We quantify the PSFs in terms of the focusing efficiency, Strehl ratio (SR), and full width of half maximum (FWHM). Here we define the focusing efficiency as the power within a 15 μm diameter circle around the PSF divided by the total input power. The SR, in its turn, is the PSF peak intensity divided by the diffraction-limited Airy disk peak intensity [25]. Both distributions are normalized to have the same energy. The SR quantifies the quality of the PSF, considered diffraction-limited for SR > 0.8 [25]. Figure 4(a)–(c) show the focusing efficiency, SR, and longitudinal FWHM as function of the angle of incidence for the first (blue solid lines) and second (black solid lines) cases, respectively. At normal incidence, both cases have FWHM ≅ 346 nm and SR ≅1 . The doublet with $f_{q}=f_{h}$ focuses about 44.17% of the incoming energy, whereas the doublet with $f_{q}=1.36f_{h}$ achieves about 87%. Therefore, at normal incidence, the SP phase profile is satisfactorily preserved for both cases since both can focus on the diffraction limit, but the former case has a much broader spectrum than the latter, causing a substantial amount of energy to be lost to diffraction, not contributing to the focusing. The broader spectrum has an even more deleterious effect at oblique incidence. Note that the SR for $f_{q}=f_{h}$ drops quickly with the angle of incidence, as shown by the black solid line in Figure 4(b), falling below the diffraction-limited spot condition (<0.8) at around 9°, resulting in an FOV of 18°. The longitudinal FWHM, by its turn, increases as the angle of incidence increases (see Figure 4(c)), reaches 387 nm at 9°. In contrast to the non-optimum doublet, all parameters (especially the focussing efficiency) are almost constant within a broad range of incidence angles for $f_{q}=1.36f_{h}$ , as indicated by the blue lines in Figure 4(a)–(c) (Recall that $f_{q}=1.36f_{h}$ was the optimum value for an NA = 0.75). In particular, the SR is greater than 0.8 for angles up to 16.5°, resulting in an FOV of 33°. At this point, the FWHM increases to 375 nm, 13 nm smaller than the non-optimum design, and at the threshold of diffraction-limited resolution.

We have now identified the optimum design conditions for a doublet system. In the next section, we build on the previous insights to obtain a universal plot for doublet designs, highlighting the trade-off between resolution (NA) and FOV, and providing the designer with the limiting conditions for particular applications.

### 2.3 Universal parameter space for doublet designs

**Figure 5:**
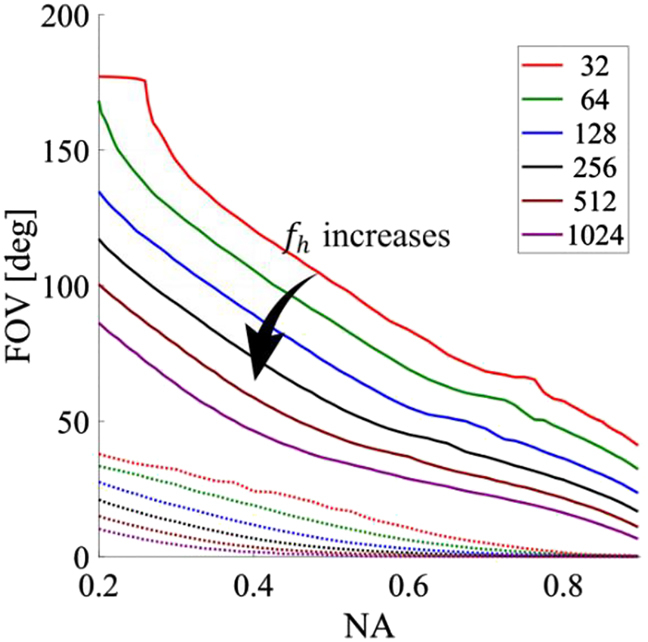
Doublet FOV (solid lines) as function of the entrance NA $\left(\sin\left(\text{atan}\left(\frac{R_{a}}{f_{h}}\right)\right)\right)$ for different focal lengths (the numbers on the plot legend refer to the focal length to operating wavelength ratio). The substrate is glass (n=1.45) . The dashed lines show the FOV of the singlet hyperbolic function.

In this final section, we calculate the NA and FOV, assuming an $f_{q}$ given by the optimum condition of Eq. (10). We assume $n_{1}=n_{3}=1$ and a spacer with $n_{2}=1.45$ . We calculate $f_{q}$ from Eq. (11) and the substrate thickness from Eq. (8) for each focal length and evaluated the doublet PSF for different incidence angles for each design, defined by the parameters ( $f_{h}$ , NA). For the sake of comparison, we also calculate the FOV (defined as twice the angle upon which the PSF Strehl ratio is smaller than 0.8) for the single-layer hyperbolic metalens using the same approach. We carry out the calculations using the angular spectrum formalism. The FOVs for the doublet (solid lines) and hyperbolic singlet (dashed lines) are shown as function of the entrance aperture NA for different focal lengths $\left(f_{h}\right)$ in Figure 5 (a map of the FOV and other parameters as function of the focal length and entrance aperture can be found in Section S6). Note that, for all focal lengths analyzed, the FOV of the doublet reduces monotonically with the NA. This is an expected behavior because, as the NA increases, the maximum spatial frequency of the SP also increases (more details are available in Section S5 of the SI), enhancing spurious diffraction within the substrate and reducing the doublet FOV. For a fixed NA, the FOV also reduces with an increasing focal length (see Figure 5). This feature is related to the difference in the centers of the quadratic phase profiles in Eq. (6): the longer the focal length, the greater the difference between the terms $d\;\tan\;\theta^{\prime }$ and $\frac{f_{q}}{n_{3}}n_{1}\;\sin\;\theta$ in Eq. (7). This feature worsens the required alignment of the quadratic phase profiles in Eq. (6) whenever the focal length increases, resulting in a narrower FOV.

Our design can achieve high FOVs while maintaining the Strehl ratio close to one (see Figure S3 on the SI). To put this feature into perspective, we compared our method with designs using the optimization approach. That is, we designed Doublet systems equivalents to the ones proposed in [16, 24], [25], [26] using our method (see Table S1 for a full description of each design). To have a fair comparison, we decided to simulate both our design and the designs proposed in each paper using the angular spectrum formalism (see Section S7 of the SI for more details). Additionally, we applied the criteria used to define the FOV in Figure 5 to all cases. For small focal lengths (smaller than 40 μm ), the optimization method resulted in very high Strehl ratios (close to one) over the whole field of view (see Table S2 in the SI). However, our method results in systems with a much higher FOV while also keeping the Strehl ratio close to one.

## 3 Conclusions

We have examined the physical principles underlying the operation of doublet metalenses. The identification of these principles has allowed us to define a universal design strategy that does not require numerical optimization and that has enabled a quantification of the fundamental trade-off between resolution and field of view in doublet systems. The rationale starts by recognizing that only the hyperbolic phase profile enables focusing at the diffraction limit, trading off FOV, and that only the quadratic phase profile achieves virtually unlimited field of view – trading off against lower resolution due to spherical aberration. The doublet takes advantage of both these features by using a quadratic metalens at the output, which provides wide FOV, and a Schmidt plate-like metalens at the input, which converts the displaced quadratic phase profile into a displaced hyperbolic one. Thus, the space between the surfaces plays a fundamental role in the optical properties of the doublet system. Additionally, we recognize that the focal length of the output quadratic phase profile can be used as a parameter to minimize the SP bandwidth and thus improve the doublet performance. We have also provided an implicit equation for the optimum quadratic phase profile focal length as function of the system NA. The analysis has allowed us to identify the fundamental limits of the doublet system, that is, the achievable range of resolution and FOV for a given focal length. For instance, a doublet metalens system with a normalized focal length of $\frac{f_{h}}{\lambda_{0}}=32$ has FOVs of 120° and 56° for NAs of 0.4 and 0.8, respectively. Similarly, a system with $\frac{f_{h}}{\lambda_{0}}=512$ can reach FOVs of 58° and 21.5° for NAs of 0.4 and 0.8, respectively. Our findings allow researchers to identify *a priori* the range of resolutions and FOVs accessible to a specific application and easily design optimized systems to achieve these fundamental limits.

## Supporting information

The following files are available free of charge. Supporting information: Angular spectrum formalism, Schmidt plate phase profile, Analysis of the propagated fields inside the doublet spacer, Impact of the spacer thickness equation on the FOV. details on the requirement of shallow phase modulation on the SP, Universal parameter space for doublet designs, Literature comparison and doublet design with an air spacer (PDF).

## Abbreviations

FOV: Field-of view
SP: Schmidt plate
NA: Numerical aperture
FWHM: Full width at half maximum
SR: Strehl ratio
PSF: Point spread function.

## Supplementary Material

Supplementary Material
