# Supplementary material for: Fundamental limits and design principles of doublet metalenses
Source: Nanophotonics. 2022 Feb 16;11(6):1187–94. doi: 10.1515/nanoph-2021-0770 (PMC11501158; doi:10.1515/nanoph-2021-0770)
Supplement: Supplementary file 1 — Supplementary Material [file j_nanoph-2021-0770_suppl.docx]

Supporting information for: Fundamental limits and design principles of doublet metalenses

Augusto Martins^1,2*^, Juntao Li^3^_,_ Ben-Hur V. Borges^1^, Thomas F. Krauss^4^ and Emiliano R. Martins^1**^

^1^São Carlos School of Engineering, Department of Electrical and Computer Engineering, University of São Paulo, Brazil, 13566-590

^2^Department of Physics, Harvard University, Cambridge, MA 02138, U.S.A.

^3^State Key Laboratory of Optoelectronic Materials and Technologies, School of Physics, Sun Yat-sen University, Guangzhou, China, 510275

^4^University of York, School of Physics, Engineering and Technology, York, UK, YO10 5DD

*Corresponding author: augustomartins@fas.harvard.edu

**Corresponding author: erm@usp.br

Supporting information

Section S1 – Angular spectrum formalism

Section S2 – Schmidt plate phase profile

Section S3 – Analysis of the propagated fields inside the doublet spacer

Section S4 – Impact of the spacer thickness equation on the FOV

Figure S1

Section S5 – Details on the requirement of shallow phase modulation on the SP

Figure S2

Figure S3

Section S6 – Universal parameter space for doublet designs

Figure S4

Figure S5

Section S7 – Literature comparison

Table S1

Table S2

Section S8 – Doublet design with an Air spacer

Figure S6

Bibliography

**Section S1 – Angular spectrum formalism**

All simulations shown in the manuscript were based on the Angular spectrum formalism [[1](#_ENREF_1)], which does not assume the paraxial approximation. We assume a background media with refractive index *n* and an operating free-space wavelength λ_0_. According to this formalism, given a field $I\left( x,y;z_{1} \right)$ on the plane $z=z_{1}$, the resulting diffracted field on a plane $z=z_{2}$ is given by:

$I\left( x,y,z_{2} \right)=\mathcal{F}^{-1}\left\{ \mathcal{F[}U\left( x,y;z_{1} \right]\left( k_{x},k_{y} \right)e^{ik_{z}\left( z_{2}-z_{1} \right)} \right\}\left( x,y \right)$ (S1.1 )

where, *I*(*x,y;z*) is the field distribution, $\mathcal{F}$ and $\mathcal{F}^{-1}$ are the bi dimensional Fourier and inverse Fourier transforms, respectively [[1](#_ENREF_1)]. Finally, *k_z_* is the z component of the wave-vector and is given by

$k_{z}=\sqrt{n^{2}k_{0}^{2}-k_{x}^{2}-k_{y}^{2}}$ (S1.2)

Where, $k_{0}=\frac{2\pi}{\lambda_{0}}$ is the wavenumber and $\lambda_{0}$ are the free space wavelength..

**Section S2 – Schmidt plate phase profile**

Although Eq. (4) is useful to highlight the physical mechanism of the Schmidt plate, it does not take into account the diffraction suffered by the beam while it propagates through the spacer. Diffraction can be conveniently accounted for using the Angular spectrum formalism (see section S1) to perform a backwards propagation from the output plane to the input plane. However, the restriction of constant field amplitude is not satisfied with this procedure and ideally one should perform an iterative process using phase retrieval algorithms such as the Gerchberg-Saxton algorithm used in computer holography [[1](#_ENREF_1)]. Nevertheless, given the requirement of a small bandwidth, we found that a single back propagation and normalization of the field input over the required entrance aperture is enough to obtain the required phase profile.

Ideally, the field reaching the output metalens should be:

$\psi\left( x,y \right)=\left\{ \begin{aligned} e^{-j\phi_{SP}\left( x,y \right)}, r\leq R_{a} \\ 0, otherwise \end{aligned} \right.$ (S2.1)

Where $\phi_{SP}$ is the phase profile given by Eq. (4) in the main text and $r\equiv\sqrt{x^{2}+y^{2}}$. Using equation (S1.1), we can back-propagate the field of equation S2.1 to obtain the SP profile as:

$\phi_{S}^{'}\left( x,y \right)=\left\{ \begin{aligned} \arg\left( \mathcal{F}^{-1}\left[ \mathcal{F}\left( \psi\left( x,y \right) \right)e^{-jk_{z}d} \right]\left( x,y \right) \right), x^{2}+y^{2}<R_{a}^{2} \\ 0, otherwise \end{aligned} \right.$ (S2.2)

Where $d$ is the substrate thickness and $k_{z}=\sqrt{n_{2}^{2}k_{0}^{2}-k_{x}^{2}-k_{y}^{2}}$. Note that the aperture radius $R_{a}$ is defined in Eq. S2.1 and the small bandwidth of $\psi$ guarantees that the backpropagated field energy is concentrated is in a region smaller $R\leq1.1R_{a}$, which justify the truncation at on $r\leq R_{a}$ in Eq. S2.2 .

**Section S3 – Analysis of the propagated fields inside the doublet spacer**

In this section we demonstrate analytically the physics described qualitatively in section 2 of the main text. All derivations are made using the Angular spectrum formalism for scalar waves (Section S1). Without loss of generality, we assume that a plane wave is incident on the doublet system at an angle $\theta$, as is represented in Fig. 1(b) of the main text. Thus, assuming setting z = 0 at the plane of the Schmidt plate:

$I_{\theta}\left( x,y,0 \right)=e^{k_{0}n_{1}y\sin\theta}e^{-\phi_{SP}\left( x,y \right)}circ\left( \frac{r}{R_{a}} \right)=e^{-k_{0}n_{1}y\sin\theta}I_{0}\left( x,y,0 \right)$ (S3.1)

Where $I_{0}\left( x,y,0 \right)\equiv e^{-\phi_{SP}\left( x,y \right)}circ\left( \frac{r}{R_{a}} \right),r\equiv\sqrt{x^{2}+y^{2}}$ is the field transmitted at normal incidence and $circ$is the circular aperture function that is given by

$circ\left( r \right)=\left\{ \begin{aligned} 1, r\geq1 \\ 0, othewise \end{aligned} \right.$ (S3.2)

The Fourier transform of $I_{0}\left( x,y,0 \right)$ is defined as $S(k_{x},k_{y})$ and is given by

$S\left( k_{x},k_{y} \right)\mathcal{\equiv F}\left[ I_{0}\left( x,y,0 \right) \right]\mathcal{=F}\left[ e^{-\phi_{SP}\left( x,y \right)} \right]\mathcal{\otimes F}\left[ circ\left( \frac{r}{R_{a}} \right) \right]=2\pi\mathcal{F}\left[ e^{-\phi_{SP}\left( x,y \right)} \right]\otimes\frac{J_{1}\left( k_{r}R_{a} \right)}{k_{r}}R_{a}$ (S3.3)

Where $\otimes$ denotes the convolution operation, $J_{1}$ is the Bessel function of first kind and order 1 and $k_{r}=\sqrt{k_{x}^{2}+k_{y}^{2}}$. Note that the function $\frac{J_{1}\left( k_{r}R_{a} \right)}{k_{r}}R_{a}$ has a bandwidth *B* given by [[1](#_ENREF_1)]:

$\frac{B}{k_{0}}=0.7\frac{\lambda_{0}}{R_{a}}=\frac{0.7}{K}$ (S3.4)

Where $K\equiv\frac{R_{a}}{\lambda_{0}}$. Since we are interested in large lenses for imaging, we can assume that the aperture is larger than 30$\mu m$, which, for visible wavelengths, amounts to $K\geq46$. Thus, from (S3.4),

$\frac{B}{k_{0}}\leq0.015$ (S3.5)

Therefore, the aperture bandwidth is too small and has little to no effect on the transmitted field. Thus, the bandwidth of the Schmidt plate is mostly defined by its phase modulation and Eq. S3.3 can be approximated as

$S\left( k_{x},k_{y} \right)\mathcal{\cong F}\left[ e^{-\phi_{SP}\left( x,y \right)} \right]\left( k_{x},k_{y} \right)$ (S3.6)

Thus, from Eq. S1.1, the field reaching the output metalens is given by

$I_{\theta}\left( x,y,d \right)=\mathcal{F}^{-1}\left\{ \mathcal{O}_{\theta}\left( k_{x},k_{y} \right) \right\}\left( x,y \right)=\mathcal{F}^{-1}\left\{ \mathcal{F}\left[ I_{\theta}\left( x,y,0 \right) \right]\left( k_{x},k_{y} \right)e^{jk_{z}d} \right\}\left( x,y \right)$ (S3.7)

Where, $\mathcal{O}_{\theta}\left( k_{x},k_{y} \right)\mathcal{\equiv F}\left[ I_{\theta}\left( x,y,0 \right) \right]\left( k_{x},k_{y} \right)e^{jk_{z}d}$ is the field spectrum at the output metalens and $k_{z}=\sqrt{n_{2}^{2}k_{0}^{2}-k_{x}^{2}-k_{y}^{2}}$. From the shifting theorem in Fourier transforms [[1](#_ENREF_1)], and Eqs. S3.1 and S3.3, $\mathcal{O}_{\theta}\left( k_{x},k_{y} \right)$is given by,

$\mathcal{O}_{\theta}\left( k_{x},k_{y} \right)=S\left( k_{x},k_{y} -k_{0}n_{1}\sin\theta\right)e^{jk_{z}d}$ (S3.8)

Under the assumption that $S\left( k_{x},k_{y} \right)$ has a small bandwidth, $k_{z}$ can be expanded in a Taylor series around the centre of $\mathcal{O}_{\theta}\left( k_{x},k_{y} \right)$, which, according to equation (S3.8) is at $\left( k_{x},k_{y} \right)=\left( 0,k_{0}n_{1}\sin\theta\right)$, and, equivalently, according to Snell’s law, at $\left( k_{x},k_{y} \right)=\left( 0,k_{0}n_{2}\sin\theta^{'} \right)$. Thus,

$k_{z}=n_{2}k_{0}\sqrt{1-\frac{\left( k_{x}^{2}+k_{y}^{2} \right)}{n_{2}^{2}k_{0}^{2}}}\cong n_{2}k_{0}\cos\theta^{'}-\left( k_{y}-k_{0}n_{2}\sin\theta^{'} \right)\tan\theta^{'}\mathcal{+E}\left[ k_{x}^{2},\left( k_{y}-k_{0}n_{2}\sin\theta^{'} \right)^{2} \right]$ (S3.9)

Where $\mathcal{E}\left( k_{x}^{2},k_{y}^{2} \right)$ is the error in the approximation of the function $k_{z}$ truncated in the first order term of Taylor series. Thus, from (S3.6), (S3.8), and (S3.9), the Fourier transform of the field reaching the output metalens is given, by:

$\mathcal{O}\left( k_{x},k_{y} \right)=S\left( k_{x},k_{y}-k_{0}n_{2}\sin\theta^{'} \right)e^{-jd\left( k_{y}-k_{0}n_{2}\sin\theta^{'} \right)\tan\theta^{'}}e^{jdn_{2}k_{0}\cos\theta^{'}}e^{jd\mathcal{E}\left[ k_{x}^{2},\left( k_{y}-k_{0}n_{2}\sin\theta^{'} \right)^{2} \right]}$ (S3.10)

The term $e^{-ik_{y}d\tan\theta^{'}}$describes the vertical displacement suffered from propagation and the term proportional to $e^{jd\mathcal{E}\left[ k_{x}^{2},\left( k_{y}-k_{0}n_{2}\sin\theta^{'} \right)^{2} \right]}$ accounts for higher order corrections to the diffraction. Additionally, note that the phase profile imposed by oblique incidence is carried on by $S\left( k_{x},k_{y}-k_{0}n_{2}\sin\theta^{'} \right)$ according to the shifting property of the Fourier transform. Therefore, from Eqs. S3.7 and 3.10, the output field in real space is given by

$I_{\theta}\left( x,y,d \right)=\mathcal{F}^{-1}\left[ S\left( k_{x},k_{y}-k_{0}n_{2}\sin\theta^{'} \right)e^{-jd\left( k_{y}-k_{0}n_{2}\sin\theta^{'} \right)\tan\theta^{'}}e^{jdn_{2}k_{0}\cos\theta^{'}}e^{j\mathcal{E}\left[ k_{x}^{2},\left( k_{y}-k_{0}n_{2}\sin\theta^{'} \right)^{2} \right]} \right]$ ( S3.11)

Applying the shifting theorem in (S3.11)

$I_{\theta}\left( x,y,d \right)=\mathcal{F}^{-1}\left[ S\left( k_{x},k_{y} \right)e^{-jk_{y}d\tan\theta^{'}}e^{j\mathcal{E}\left[ k_{x}^{2},k_{y}^{2} \right]} \right]e^{jyk_{0}n_{2}\sin\theta^{'}}e^{jdn_{2}k_{0}\cos\theta^{'}}$ ( S3.12)

From the convolution theorem, Eq. S3.6 and applying the shifting theorem for the linear phase $e^{-jk_{y}d\tan\theta^{'}}$ we obtain the field reaching the output metalens:

$I_{\theta}\left( x,y,d \right)=I_{0}\left( x,y-d\tan\theta^{'},0 \right)\otimes\mathcal{F}^{-1}\left[ e^{j\mathcal{E}\left[ k_{x}^{2},k_{y}^{2} \right]} \right]e^{jyk_{0}n_{2}\sin\theta^{'}}e^{jdn_{2}k_{0}\cos\theta^{'}}$ (S3.13)

As discussed qualitatively in the main text, we have showed mathematically that, under the approximation of a Schmidt plate with small bandwidth, the field in the output is given by:

- $I_{0}\left( x,y-d\tan\theta^{'},0 \right)$: displaced input field
- $\mathcal{F}^{-1}\left[ e^{j\mathcal{E}\left[ k_{x}^{2},k_{y}^{2} \right]} \right]$: Diffraction in the substrate, which can be neglected if the spectrum of the Schmidt plate is very small.
- $e^{jyk_{0}n_{2}\sin\theta^{'}}$: linear phase profile from the incidence
- $e^{jn_{2}k_{0}\cos\theta^{'}}$: constant phase term

Finally, including the contribution of the quadratic metalens to the field modulation, the output field is given by

$$I_{out}\left( x,y \right)=I_{\theta}\left( x,y,d \right)e^{j\phi_{q}\left( x,y \right)}=$$

$=I_{0}\left( x,y-d\tan\theta^{'},0 \right)\otimes\mathcal{F}^{-1}\left[ e^{j\mathcal{E}\left[ k_{x}^{2},k_{y}^{2} \right]} \right]e^{j\phi_{q}\left( x,y-\frac{f_{q}}{n_{3}}n_{1}\sin\theta\right)}e^{jn_{2}k_{0}\left( d\cos\theta^{'}+\frac{k_{0}n_{2}}{2n_{3}}f_{q}\sin^{2} \theta^{'} \right)}$ (S3.14)

Where we used equation (5) from the main text. Equation (S3.14) is the rigorous counterpart to equation (7) of the main.

**Section S4 – Impact of the spacer thickness equation on the FOV**

Here we address explicitly the question of how much Equation 8 can depart from Equation 9 within the system’s FOV. To answer this question, we define the parameter δ as the difference between the propagation and quadratic phases for an angle of incidence *θ*:

$\delta=d\left| \tan\theta^{'}-\sin\theta^{'} \right|$ (S4.1)

Where *θ’* is the angle of refraction related to the incoming angle *θ* through Snell’s law.

The FOV can now be associated with a maximum misalignment *δ*=*ϵ*.Thus, the maximum angle of incidence θ defining the FOV must satisfy

$\left| \tan\theta^{'}-\sin\theta^{'} \right|=\frac{\epsilon}{d}$ (S4.2)

The function $g\left( \theta^{'} \right)\equiv\left| \tan\theta^{'}-\sin\theta^{'} \right|$ is shown in Figure S1 (a). As expected, it is a monotonically increasing function. Its maximum value, that is, the tolerance $\epsilon$, can be found by identifying the maximum angle of incidence from the FOV of the system (recall that the FOV itself is defined in terms of the Strehl ratio).


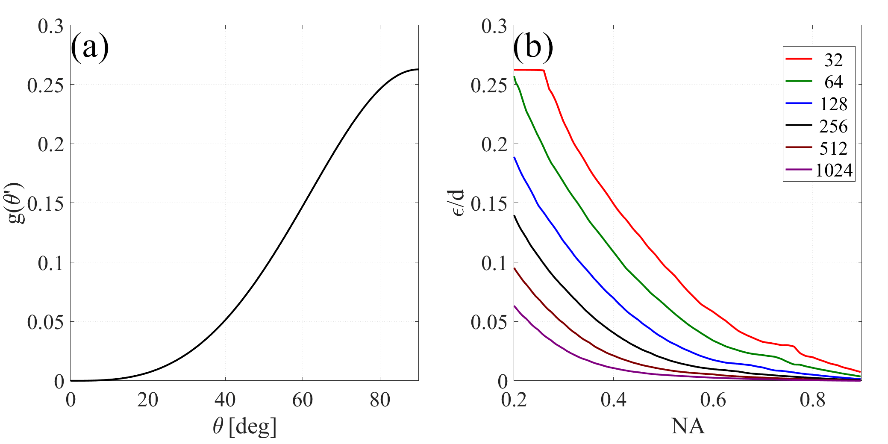


Fig S1 – (a) Shows $g\left( \theta^{'}(\theta) \right)=\left| \tan\theta^{'}-\sin\theta^{'} \right|$, where θ is the angle of incidence. (b) shows the normalized error approximation $\frac{\epsilon}{d}$ as function of the entrance NA $\left( \sin\left( \mathrm{atan} \left( \frac{R_{a}}{f_{h}} \right) \right) \right)$ for different focal lengths (the numbers on the plot legend refer to the focal length to operating wavelength ratio). The substrate is glass $\left( n=1.45 \right)$.

Figure S1 (b) shows $\frac{\epsilon}{d}$ and it closely resembles the FOV plot of Figure 5 of the main text: the tolerance becomes smaller as the NA or focal length increase, which directly reduces the FOV. Thus, as correctly perceived by the reviewer, large NAs entail lower tolerances, and consequently smaller FOVs. The tolerance, however, is better for lower focal lengths, because these entail shorter spacers, thus contributing to a lower discrepancy between the optical propagation displacement and the quadratic phase displacement (that is, lower $\frac{\epsilon}{d}$ as shown in (S4.2)).

**Section S5 – Details on the requirement of shallow phase modulation on the SP**

In this section we obtain equation (11) of the main text, which is an implicit equation for the parameter $\alpha=\frac{f_{q}}{f_{h}}$ that minimizes $\max\left| \frac{\partial\phi_{SP}}{\partial r} \right|$ for a given numerical aperture. To begin with, let us obtain the expression for the spatial frequency equation $\frac{\partial\phi_{SP}}{\partial r}$. The approximated SP phase profile is given by

$\phi_{SP}\left( r \right)=-k_{0}n_{3}\left( \sqrt{r^{2}+f_{h}^{2}}-\frac{r^{2}}{2f_{q}} \right) ,0\leq r\leq R_{a}$ (S5.1)

Thus, its radial derivative is given by

$\frac{\partial\phi_{SP}}{\partial r}=-k_{0}n_{3}\left( \frac{r}{\sqrt{r^{2}+f_{h}^{2}}}-\frac{r}{f_{q}} \right)=-\frac{k_{0}n_{3}r}{f_{q}\sqrt{r^{2}+f_{h}^{2}}}\left( f_{q}-\sqrt{r^{2}+f_{h}^{2}} \right)0\leq r\leq R_{a}$ (S5.2)

Equation (3) is a continuous function defined in a closed interval. Therefore, its maximum absolute value lies either in the domain borders $(r=0$ and $r=R_{a}$) or in a local maximum within the interval. We now evaluate $\frac{\partial\phi_{SP}}{\partial r}$ for all these cases and compare the results.

- $r=0$

$\frac{\partial\phi_{SP}}{\partial r}\left. \right|_{r=0}=0$ (S5.3)

Thus, the phase gradient is clearly not maximum at $r=0$

- $r=R$

$M_{R_{a}}\left( \alpha\right)\equiv\left| \frac{\partial\phi_{SP}}{\partial r}\left. \right|_{r=R_{a}} \right|=\left| -k_{0}n_{3}\left( \frac{R_{a}}{\sqrt{R_{a}^{2}+f_{h}^{2}}}-\frac{R_{a}}{f_{q}} \right) \right|=k_{0}n_{3}\frac{F}{\sqrt{F^{2}+1}}\frac{1}{\alpha} \left| \sqrt{F^{2}+1}-\alpha\right|$ (S5.4)

where $F\equiv R_{a}/f_{h}$.

- Local maximum

$\frac{\partial^{2}\phi}{\partial r^{2}}=0=-k_{0}n_{3}\left( \frac{f_{h}^{2}}{\left( \rho^{2}+f_{h}^{2} \right)^{\frac{3}{2}}}-\frac{1}{f_{q}} \right)$ (S5.5)

From Eq. (S5.5)

$\frac{\rho^{2}}{f_{h}^{2}}=\left( \frac{f_{q}}{f_{h}} \right)^{\frac{2}{3}}-1=\alpha^{\frac{2}{3}}-1$ (S5.6)

Note that, since the radius is defined in the interval $0\leq\rho\leq R_{a}$ then, according to equation S5.6, the focus ratio is defined in the range $1\leq\alpha\leq\left( F^{2}+1 \right)^{\frac{3}{2}}$

Substituting equation (S5.6) on (S5.2), we find that the phase gradient modulus at the local maximum is given by

$M_{e}\left( \alpha\right)\equiv\left| \frac{\partial\phi}{\partial r}\left. \right|_{r=\rho} \right|=\left| -k_{0}n_{3}\frac{\left( \alpha^{\frac{2}{3}}-1 \right)^{\frac{3}{2}}}{\alpha} \right|=k_{0}n_{3}\frac{\left( \alpha^{\frac{2}{3}}-1 \right)^{\frac{3}{2}}}{\alpha}$ (S5.7)

The phase gradient at the local maximum ($M_{e}\left( \alpha\right)$) is a monotonically increasing function of $\alpha$ $\left( \frac{dM_{e}\left( \alpha\right)}{d\alpha}\geq0 \right)$ and it is equal to 0 at $\alpha=1$ according to Eq. S5.7. The phase gradient modulus at the edge $\left( M_{R_{a}}\left( \alpha\right) \right)$, in contrast, is monotonically decrescent for $\alpha<\sqrt{F^{2}+1}$ and crescent for $\alpha>\sqrt{F^{2}+1}$ (at $\alpha=\sqrt{F^{2}+1}$, the function has a kink from the modulus operator and its derivative is not defined). Additionally, $M_{R_{a}}\left( 1 \right)=k_{0}n_{3}\frac{F}{\sqrt{F^{2}+1}} \left| \sqrt{F^{2}+1}-1 \right|\geq0$. To illustrate these properties, an example of $M_{e}\left( \alpha\right)$ and $M_{R_{a}}\left( \alpha\right)$ assuming *F=1*, is shown in Fig. S2, where $K\equiv\sqrt{F^{2}+1}$


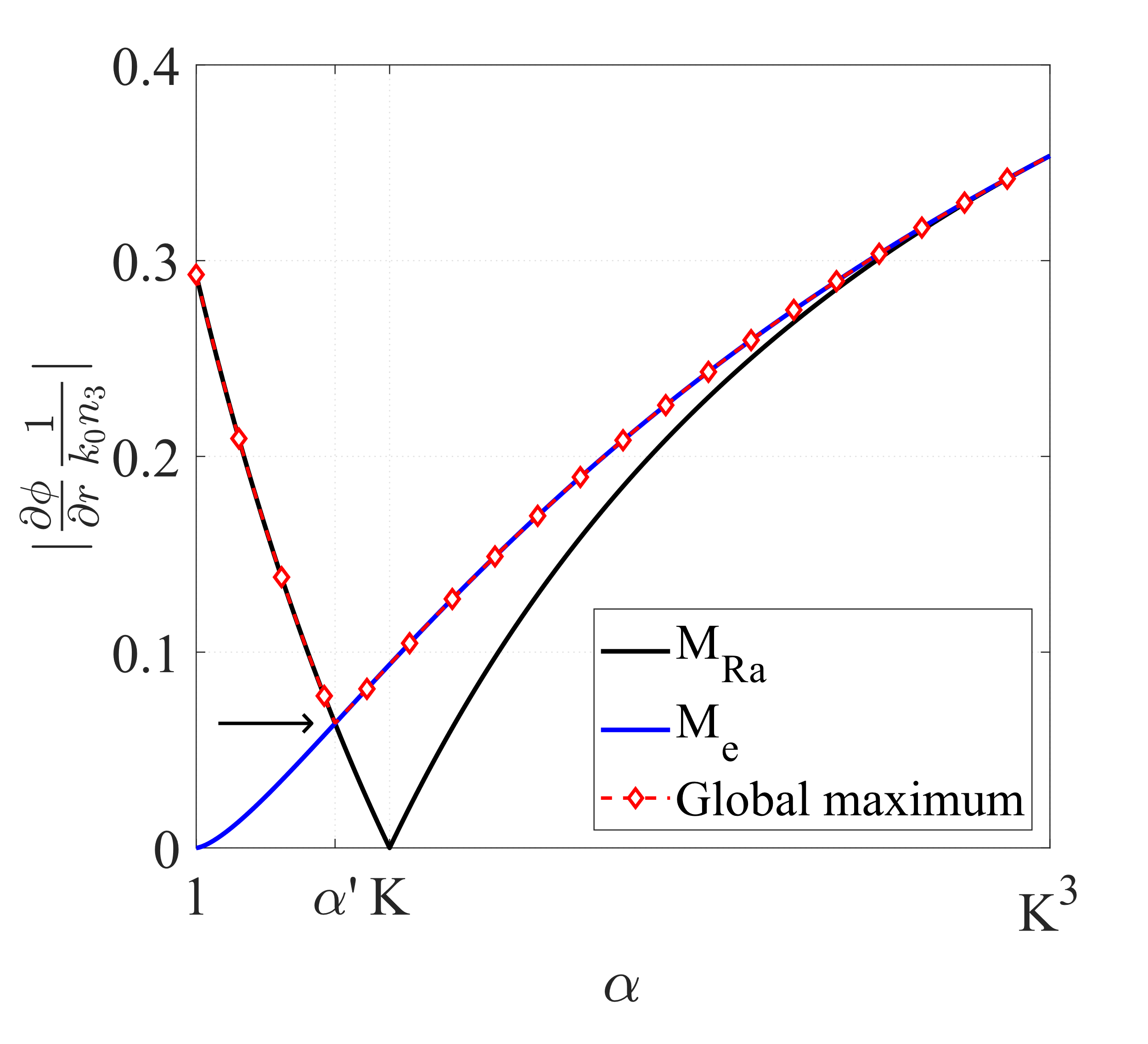


Fig S2 – Phase gradient modulus at the local border $(M_{Ra}$, black curve) and at the local maximum ($M_{e}$, blue curve) as function of $\alpha$ for $F=1$. It is also shown the global maximum of the phase gradient modulus. We defined $K\equiv\sqrt{F^{2}+1}$ and $\alpha^{'}$ is the point that minimizes the global maximum.

As $\alpha$ increases from 1, $M_{e}\left( \alpha\right)$ increases from zero whereas $M_{R_{a}}\left( \alpha\right)$, which is the global maximum in this condition, decreases (see Fig S2). Since $M_{R_{a}}\left( \sqrt{F^{2}+1} \right)=0,$ these functions must cross at some point in the interval $1\leq\alpha^{'}\leq\sqrt{F^{2}+1}$, as illustrated in Fig. S2. After this point, $M_{e}\left( \alpha\right)$ becomes the global maximum, which increases monotonically with $\alpha$, as represented in Fig. S2. Thus, the phase gradient modulus is minimized at the first point where its local extrema equals its value at the border, as indicated by the arrow in Fig S2. In other words, we must solve the equation

$M_{e}\left( \alpha^{'} \right)=M_{R_{a}}\left( \alpha^{'} \right), 1\leq\alpha^{'}<\sqrt{F^{2}+1}$ (S5.8)

Thus, from (S5.4), (S5.7) and (S5.8)

$\frac{F}{\sqrt{F^{2}+1}}\frac{1}{\alpha} \left( \sqrt{F^{2}+1}-\alpha\right)=\frac{\left( \alpha^{\frac{2}{3}}-1 \right)^{\frac{3}{2}}}{\alpha}$ $, 1\leq\alpha<\sqrt{F^{2}+1}$ (S5.9)

Therefore, we can find the following implicit equation for $\alpha$ (the inverse is a cumbersome function):

$F\left( \alpha\right)=\left( \alpha^{\frac{2}{3}}-1 \right)^{\frac{1}{2}}\left[ \alpha^{\frac{2}{3}}+\left( \alpha^{\frac{4}{3}}+1-\alpha^{\frac{2}{3}} \right)^{\frac{1}{2}} \right]$, $\alpha\geq1$ (S5.10)

Note that $F\left( \alpha\right)$ is a bijective function and can be inversed, which means that there is only one possible choice of $\alpha$ for a given $F\left( \alpha\right)$. Unfortunately, there is no simple analytical form for $\alpha\left( F \right)$ and it is easier to analyse the function graphically using (S5.10) as was done in the main text.

Finally, from (S5.4) and (S5.10), the maximum phase gradient modulus is given by:

$M_{R_{a}}\left( NA \right)\equiv k_{0}n_{3} NA\left| \left( 1-\frac{1}{\alpha\left( NA \right)\sqrt{1-NA^{2}}} \right) \right|$ (S5.11)

where, $NA\equiv\frac{F}{\sqrt{F^{2}+1}}$.

According to (S5.11), the maximum spatial frequency is also independent of the doublet focal length for a fixed f-number (NA). Note that we wrote $\alpha$ as function of NA, which can be done since $F\left( \alpha\right)$ and $NA\left( F\left( \alpha\right) \right)$ are invertible functions. Fig. S3 shows $\left| \max\frac{\partial\phi}{\partial r} \right|\frac{1}{k_{0}n_{3}}$ as function of NA highlighting how quickly it grows concomitantly to the NA. Therefore, we expect that for a fixed focal length the doublet FOV reduces as its NA increases since the SP spectrum bandwidth increases, which enhances spurious diffraction in the substrate. Adittionally, for a fixed NA, as the focal length and, consequently, the spacer thickness, increases (Eq. (8) in the main text), which deteriorates the phase alignment in the output lens, making the paraxial approximation of Eq. (7) of the main text worse. Thus, the FOV is expected to be reduced as the focal length is increased for a fixed *F*.


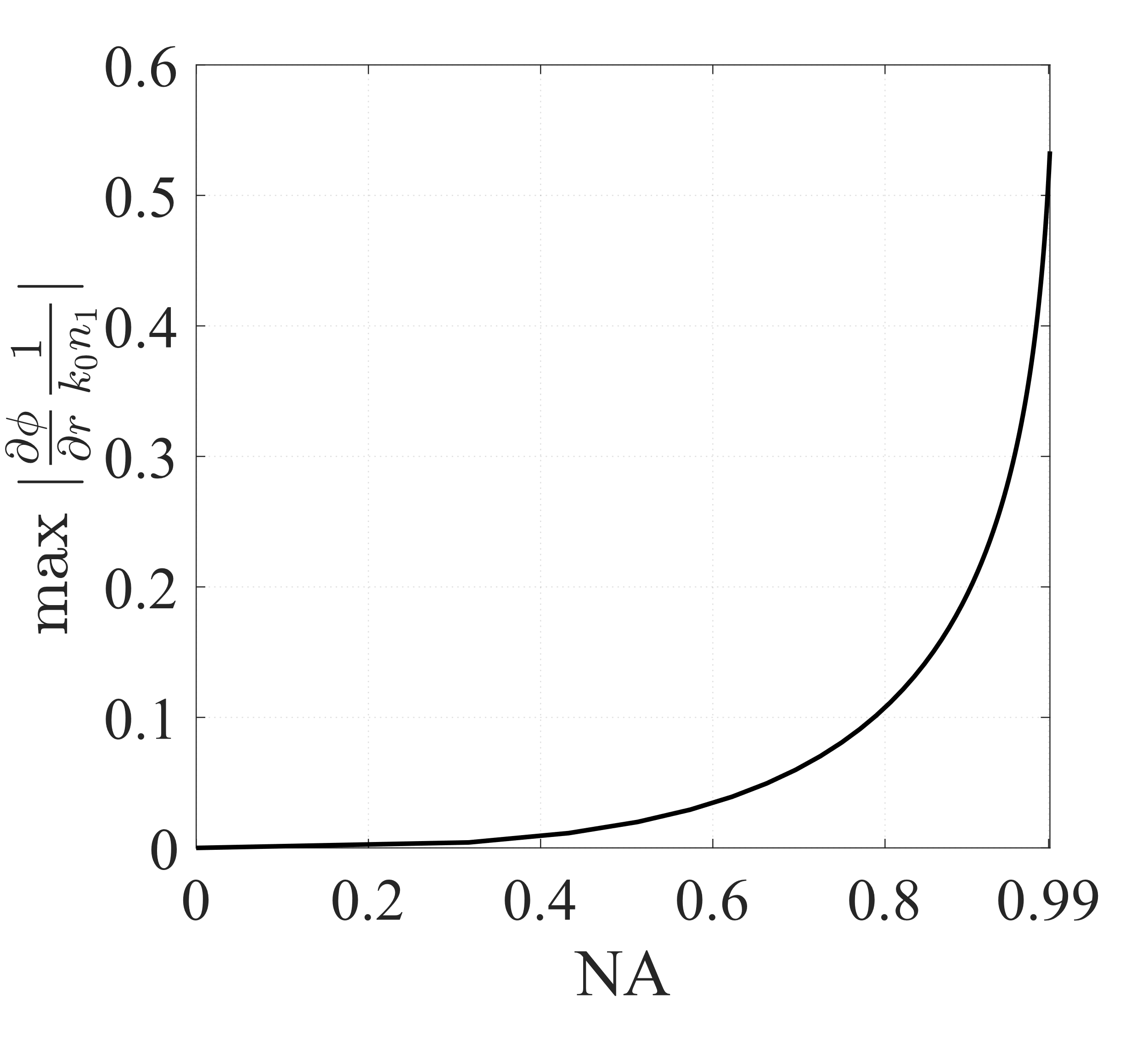


Fig S3 – Maximum phase gradient for the as function of NA (Eq. S5.11).

**Section S6 – Universal parameter space for doublet designs**


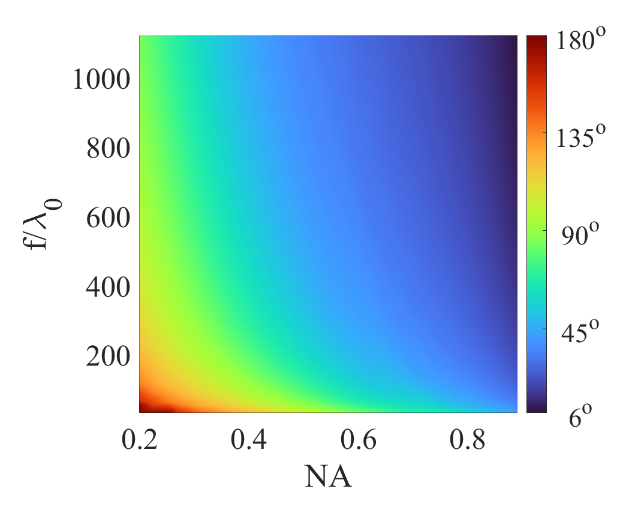


Fig S4 – Doublet FOV map as function of the entrance NA ($\sin\left( \mathrm{atan} \left( \frac{R_{a}}{f_{h}} \right) \right)$ ) and focal lengths to operating wavelength ratio. The substrate is glass (n=1.45).

**
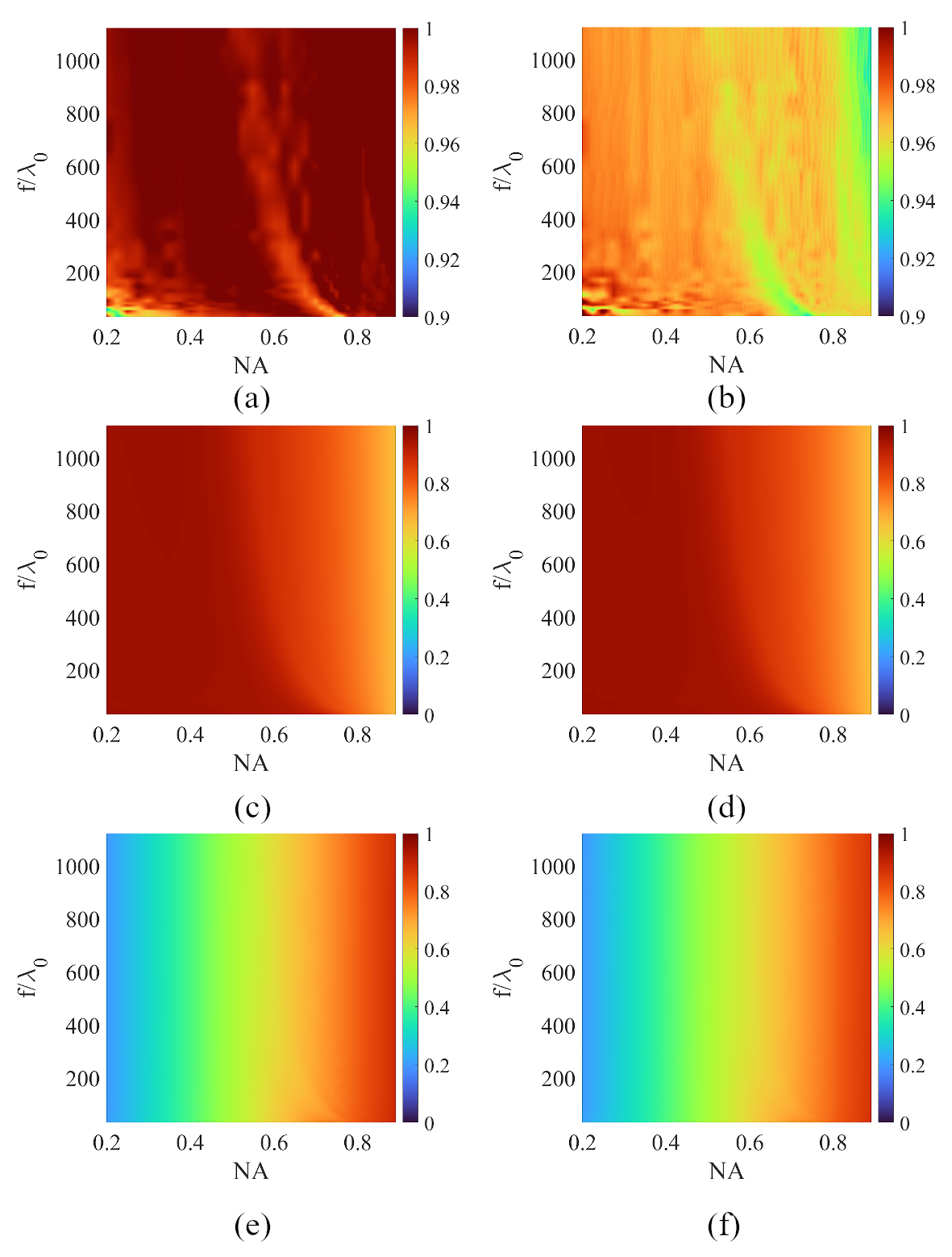
**

Fig S5 – (a), (c) and (e) show the Strehl ratio, focusing efficiency and PSF NA $\left( \frac{0.51\lambda_{0}}{FWHM} \right)$, respectively at normal incidence as function of the entrance NA ($\sin\left( \mathrm{atan} \left( \frac{R_{a}}{f_{h}} \right) \right)$ ) and focal length to operating wavelength ratio. (b), (d) and (f) show the same parameters but averaged within the FOV region. The substrate is glass (n=1.45).

**Section S7 – Literature comparison**

To compare our method with the optimization approach, we applied it to doublet designs that have been previously proposed in the literature, all of them based on numerical optimisation. Table S1 lists the references selected for this analysis. Ref [[2](#_ENREF_2)] uses a doublet with input aperture of 400 $\mu m$ designed to operate in the near infrared (850 nm). The spacer is made of silica and is 1 mm thick. The two metasurfaces are made of 600 nm thick amorphous silicon nanoposts. Ref [[3](#_ENREF_3)] shows a doublet with input aperture of 156.4 $\mu m$ designed to operate in the visible range (532 nm). The spacer is made of 0.5 mm thick glass and the metasurfaces are made of 600 nm thick TiO2 nanoposts. Ref [[4](#_ENREF_4)] shows three different doublet designs optimized to operate at the 473 nm, 532nm and 632.8 nm. All of them use a 20 $\mu m$ thick glass spacer and the input apertures are all equal to 15 $\mu m$. The metasurfaces are also made of 600 nm thick TiO2 nanoposts. Finally, ref [[5](#_ENREF_5)] shows a doublet with input aperture of 20 $\mu m$ designed to operate in the visible range (632 nm). The spacer is made of 30 $\mu m$ thick glass and the metasurfaces are made of 600 nm thick TiO2 nanoposts.

Table S1 –Doublet systems proposed in the literature using the optimization approach

| Ref | Year | λ_0_ (nm) | Layers | FoV (degs) | NA | Focal length (um) | Phase profile output | Phase profile Input |
| --- | --- | --- | --- | --- | --- | --- | --- | --- |
| [[2](#_ENREF_2)] | 2016 | 850 | 2 | 60 | 0.48 | 717 | Hyperbolic | Poly (5terms) |
| [[3](#_ENREF_3)] | 2017 | 532 | 2 | 50 | 0.44 | 342.5 | Hyp+Poly (5 terms) | Poly (5terms) |
| [[4](#_ENREF_4)] | 2020 | RGB | 2 | 40 | 0.38 | 40 | Poly (3terms) | Poly (3terms) |
| [[5](#_ENREF_5)] | 2021 | 632 | 2 | 50 | 0.44 | 21.5 | Poly (5terms) | Poly (5terms) |

Since the references listed in Table S1 do not agree with each other on the criteria to define the FOV, we decided to simulate their PSFs as function of the angle of incidence using the Angular spectrum formalism and then apply the same criteria used in the main text. Additionally, we designed equivalent doublet systems using our method. According to our simulations, the focal lengths of the designs proposed refs [[2](#_ENREF_2)] and [[3](#_ENREF_3)] are 656 $\mu m$ and 320 $\mu m$, respectively, which are slightly smaller than the values that they claim. To avoid inconsistencies, we designed our equivalent system using the values from our simulations. Table S2 summarizes the main PSF parameters obtained for each case. For example, the doublet system proposed in [[2](#_ENREF_2)] has a FOV of 50.6° with a Strehl ratio of 1.0 at normal incidence and an average of 0.9 within the FOV range. The equivalent system designed with our approach is capable of reaching a FOV of 37.4° with Strehl ratio of 1 at normal incidence and an average of 0.96 within the FOV range.

Table S2 –Doublet parameters obtained for each doublet system listed in Table S1. “Optim.” refers to the results of the optimized designs proposed in each paper.

|  | [[5](#_ENREF_5)] | | [[4](#_ENREF_4)] | | | | | | [[3](#_ENREF_3)] | | [[2](#_ENREF_2)] | |
| --- | --- | --- | --- | --- | --- | --- | --- | --- | --- | --- | --- | --- |
|  | Optim. | This paper | Optim. | This paper | Optim. | This paper | Optim. | This paper | Optim. | This paper | Optim. | This paper |
| $\frac{f}{\lambda_{0}}$ | 34.0 | | 63.3 | | 75.2 | | 84.6 | | 602.0 | | 770.6 | |
| $\lambda_{0}$ (nm) | 632 | | 473 | | 532 | | 632 | | 532 | | 850 | |
| $NA$ | 0.42 | | 0.35 | | | | | | 0.44 | | 0.52 | |
| PSF NA ($\frac{0.51\lambda_{0}}{FWHM}$) | 0.49 | 0.43 | 0.47 | 0.35 | 0.47 | 0.35 | 0.48 | 0.35 | 0.45 | 0.44 | 0.55 | 0.52 |
| FOV | 90.1 | 113.1 | 20.8 | 115.5 | 17.1 | 109.2 | 14.6 | 108.1 | 25.0 | 48.5 | 50.6 | 37.4 |
| SR1  (normal \| av.*) | 1.0 \| 0.98 | 0.98 \| 0.97 | 0.95 \| 0.92 | 0.98\| 0.98 | 0.94\| 0.90 | 0.98 \| 0.97 | 0.91 \| 0.88 | 0.99 \| 0.98 | 0.87 \| 0.85 | 1.00 \| 0.97 | 1.00 \| 0.90 | 1.00 \| 0.96 |

*The averages were calculated for angles in the FOV range.

**Section S8 – Doublet design with an Air spacer**

Figure S6 shows a performance comparison between air and glass spacer. The performance of the higher index spacer (glass - blue curve) is superior to the performance of the low index spacer (air – black curve). This is a consequence of the lower angles of refraction occasioned by the higher index. These lower angles contribute to a better paraxial approximation (the approximation of equation 9 in the main manuscript).


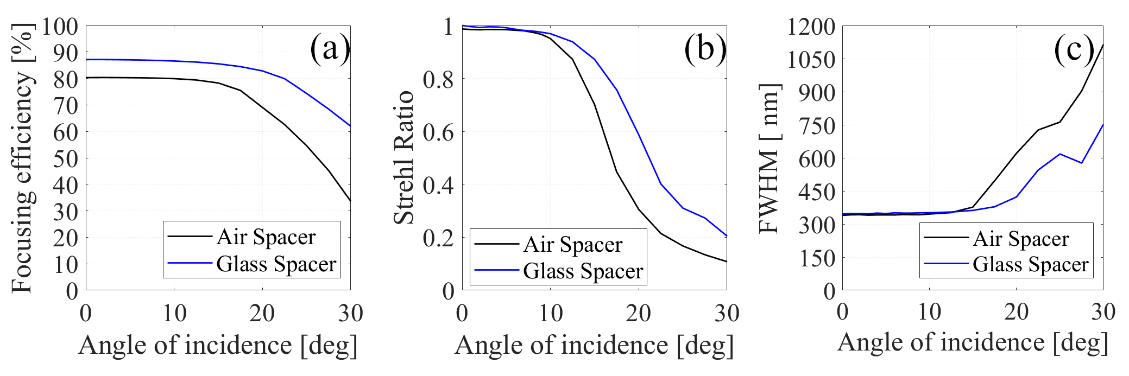


Fig S6 – Doublet point spread function parameters as function of the angle of incidence for design using an air spacer (solid black lines) and a glass spacer (blue solid lines). (a) Focusing efficiency, (b) Strehl ratio, and (c) FWHM. The operating wavelength is 532 nm, *f_h_*=100 $\mu m$ and NA = 0.75.

**Bibliography**

[1] J. W. Goodman, *Introduction to Fourier Optics* vol. 2: Roberts & Company Publishers, 2005.

[2] A. Arbabi, E. Arbabi, S. M. Kamali, Y. Horie, S. Han, and A. Faraon, "Miniature optical planar camera based on a wide-angle metasurface doublet corrected for monochromatic aberrations," *Nature Communications,* vol. 7, p. 13682, 2016.

[3] B. Groever, W. T. Chen, and F. Capasso, "Meta-Lens Doublet in the Visible Region," *Nano Letters,* vol. 17, pp. 4902-4907, 2017/08/09 2017.

[4] D. Tang, L. Chen, J. Liu, and X. Zhang, "Achromatic metasurface doublet with a wide incident angle for light focusing," *Optics Express,* vol. 28, pp. 12209-12218, 2020/04/13 2020.

[5] Z. Li, C. Wang, Y. Wang, X. Lu, Y. Guo, X. Li, X. Ma, M. Pu, and X. Luo, "Super-oscillatory metasurface doublet for sub-diffraction focusing with a large incident angle," *Optics Express,* vol. 29, pp. 9991-9999, 2021/03/29 2021.
